# The Role of Inflammation in the Pathogenesis of Osteoarthritis

**DOI:** 10.1155/2020/8293921

**Published:** 2020-03-03

**Authors:** Yoke Yue Chow, Kok-Yong Chin

**Affiliations:** Department of Pharmacology, Faculty of Medicine, Universiti Kebangsaan Malaysia, Cheras 56000, Malaysia

## Abstract

A joint is the point of connection between two bones in our body. Inflammation of the joint leads to several diseases, including osteoarthritis, which is the concern of this review. Osteoarthritis is a common chronic debilitating joint disease mainly affecting the elderly. Several studies showed that inflammation triggered by factors like biomechanical stress is involved in the development of osteoarthritis. This stimulates the release of early-stage inflammatory cytokines like interleukin-1 beta (IL-1*β*), which in turn induces the activation of signaling pathways, such as nuclear factor kappa-light-chain-enhancer of activated B cells (NF-*κ*B), phosphoinositide 3-kinase/protein kinase B (PI3K/AKT), and mitogen-activated protein kinase (MAPK). These events, in turn, generate more inflammatory molecules. Subsequently, collagenase like matrix metalloproteinases-13 (MMP-13) will degrade the extracellular matrix. As a result, anatomical and physiological functions of the joint are altered. This review is aimed at summarizing the previous studies highlighting the involvement of inflammation in the pathogenesis of osteoarthritis.

## 1. Introduction

Osteoarthritis or degenerative arthritis is a public health issue in an aging society. It is a chronic musculoskeletal disorder of the movable joints, such as knee and hip joints [1]. Osteoarthritis affects around 250 million people around the world, the majority of which are the elderly [2]. Changes in the joint tissues during aging can contribute to the development of osteoarthritis. For instance, an increase in cells manifesting senescent secretory phenotype leads to enhanced production of cytokines and matrix metalloproteinases (MMPs) in the joint environment [3]. Furthermore, reduced growth factors and the responsiveness of chondrocytes will cause less matrix synthesis and repair [3]. Other than aging, environmental, biomechanical, and biochemical factors can also contribute to the initiation of osteoarthritis. Osteoarthritis affects the entire structures of the joints, including articular cartilage, subchondral bone, meniscus, synovial membrane, and infrapatellar fat pad (IFP). The common structural characteristics of osteoarthritis are cartilage degradation, subchondral bone remodeling, osteophyte formation, and changes in the synovium and joint capsule [4].

Patients with osteoarthritis have typical clinical symptoms, such as severe joint pain, stiffness, and significantly reduced mobility, leading to decreased productivity and quality of life among the patients, as well as increased socioeconomic burden to the patients and the society [5]. As the prevalence of osteoarthritis increases with age, the aging population worldwide makes this disease a nonnegligible issue [6]. Current therapies for osteoarthritis are limited to symptom-relieving drugs and total knee arthroplasty for severe cases. Drugs addressing the underlying biological causes of osteoarthritis are not available in the market currently [7].

The involvement of immune cells in the development and progression of osteoarthritis has been highlighted in recent studies [8]. Inflammatory components, such as cytokines and chemokines, are produced by chondrocytes and synoviocytes in the joints of patients with osteoarthritis. Synovial fibroblasts are also a source of proinflammatory cytokines and matrix-degrading enzymes under osteoarthritis condition [9]. Moreover, IFP has been shown to contain a significant amount of immune cells like macrophage and T cells. As a result, IFP acts as a site of inflammatory mediators in osteoarthritic knee [10]. These inflammatory mediators alter cell signaling pathways, gene expression, and behavior of joint tissue [11]. The changes in cellular signal transduction lead to enhanced activation of the inflammatory pathway. Thus, more inflammatory compounds and enzymes are released. As a result, anatomical and physiological functions of the joint are altered [12].

This review is aimed at summarizing the recent clinical and preclinical studies performed previously to investigate the relationship between joint inflammation and the pathogenesis of osteoarthritis. The mechanism by which inflammation contributes to the pathogenesis of osteoarthritis will also be discussed.

## 2. Literature Search

A literature search on original articles written in English and published between 2014 and 2019 was performed using Scopus and PubMed database with the string: (osteoarthritis OR chondrocytes) AND inflammation. Both preclinical and clinical studies were included in this review. The search showed studies on human, animal, and cell lines investigating the involvement of inflammation in osteoarthritis by determining the differential expressions of genes, inflammatory components, and enzymes. Samples collected in human studies include synovial fluid and articular cartilage, from patients who underwent total knee arthroplasty, as well as blood (Table 1). For animal studies, agents, such as interleukin-1 beta (IL-1*β*) and monosodium iodoacetate (MIA), were used to induce osteoarthritis in the animal models (Table 2). Samples like articular cartilage and synovial tissue were taken after the treatment period. Agents such as IL-1*β* and tumor necrosis factor-alpha (TNF-*α*) were applied on cell lines to trigger inflammation (Table 3).

## 3. The Role of Immune Cells in Osteoarthritis

Immune cells like activated neutrophils and macrophages can secrete cytokines, such as IL-6 and IL-1*β*, which amplify the inflammatory process in osteoarthritis [13]. Increased infiltration of leukocytes (macrophages, T-lymphocytes, B-lymphocytes, and neutrophils) in the synovium, particularly within the subintimal layer, is a characteristic of osteoarthritis [14]. Shan et al. reported elevated PD1+CXCR5+CD4+ T cells, ICOS+CXCR5+CD4+ T cells, and IL 21+CXCR5+CD4+ T cells in peripheral blood of patients with osteoarthritis [15]. CD4+ T cell is the T helper cell (Th cell) which may induce inflammation in the early stage of osteoarthritis. C-X-C chemokine receptor type 5 (CXCR5), inducible costimulator (ICOS), and programmed cell death 1 (PD-1) are known to be expressed by the Th cell [15].

Lymphocyte activation gene-3 (LAG-3+) regulatory T cells (Treg cells) have also been shown to increase in osteoarthritis [16]. Treg cells act as an immunoregulator in many inflammatory diseases [17]. It regulates the secretion of anti-inflammatory cytokines and expression of cytokine receptors [17]. Evidence showed that the response of Treg cells decreased in osteoarthritis in concurrent with an increase of LAG-3 expression in osteoarthritis. It has been postulated that LAG-3 molecules can reduce Treg function and boost inflammation [16].

CD3+ T cells are revealed as the predominant immune cells in IFP of dogs with canine cruciate ligament disease, which is associated with osteoarthritis, followed by CD14+ macrophages [18]. Both cell types can produce various cytokines such as IL-1*β* and IL-6 when activated. Immune cells and inflammatory mediators secreted in IFP will interact with other joint tissues, which can promote the pathological process of osteoarthritis [10]. At the same time, cartilage and the synovium are also shown to modulate the IFP. IL-1*β* stimulates increased proinflammatory cytokine secretion by IFP [19], suggesting that a cross-talk happens between IFP and joint tissues.

## 4. The Role of Cytokines in Osteoarthritis

Cytokines secreted by the immune cells are the main players of any inflammatory conditions, including osteoarthritis [7]. Proinflammatory cytokines, such as IL-1*β* and TNF-*α*, are among the mediators secreted in early osteoarthritis [20–23]. IL-1*β* and TNF-*α* drive the inflammatory cascade independently or in collaboration with other cytokines [24]. They are produced by activated chondrocytes, synoviocytes, and mononuclear cells [25]. TNF-*α* and IL-1*β* have been used to trigger inflammation in chondrocyte and synoviocyte culture. Upon stimulation, the cells release IL-6 [26], IL-8 [27], IL-10 [28], IL-1*β* [29], and TNF-*α* [28]. Similar cytokine profile was increased in animal models of osteoarthritis [18, 30–34].

IL-1*β* is involved in a series of cellular activities, such as cell proliferation, differentiation, and apoptosis. It interferes with the production of essential structural proteins, including collagen type II and aggrecan, by influencing the activity of chondrocytes in the joint. Moreover, IL-1*β* affects MMPs' synthesis by chondrocytes, including MMP-1 and MMP-13, which, in turn, destroy the articular cartilage [35]. IL-1*β* was also shown to induce the production of reactive oxygen species, for example, nitric oxide (NO) [36]. Since IL-1*β* has been proven to play a significant role in the pathogenesis of osteoarthritis, it is commonly used to induce an in vitro osteoarthritis model in chondrocytes [37]. It stimulates expression of TNF-*α* and surface expression of TNF receptor (TNFR) in chondrocytes [38]. Binding of TNF-*α* to TNFR causes signal transduction and activates TNF receptor-associated factor 2 (TRAF2). TRAF2 will activate the nuclear factor kappa-light-chain-enhancer of activated B cell (NF-*κ*B) signaling pathway involved in inflammatory diseases.

A study conducted among patients with osteoarthritis showed that IL-1*β*, IL-6, IL-8, IL-18, IL-17, IL-22, and transforming growth factor-beta 1 (TGF*β*1) were increased in the inflamed synovium tissues compared to the noninflamed tissues [14]. IL-17 induces the release of IL-6, IL-8, and TNF-*α* by synovial fibroblasts and chondrocytes, leading to inflammation and cartilage breakdown [39]. It is secreted by T helper 17 cell (Th17), mast cell, and myeloid cell. Other than that, IL-17 promotes the recruitment and activation of neutrophils, which are the initial cell types recruited to the inflammation sites [40]. Activated neutrophils synthesize several inflammatory factors, which regulate the inflammation process in osteoarthritis. IL-17 has been shown to be present in the synovial fluid of a subset of patients with end-stage osteoarthritis [41]. Increased IL-17 and IL-22 levels are also detected in the synovial fluid of temporomandibular joint of patients with osteoarthritis [42]. The increase of these two cytokines is associated with the elevation of receptor activator of nuclear factor kappa-*Β* ligand (RANKL), which induces the differentiation of osteoclasts and resorption of subchondral bone, a layer of bone beneath the cartilage in joint [42]. IL-22 stimulates the proliferation of synovial cells and enhances the expression of MMPs in fibroblast-like synoviocytes (FLS) [43].

Interleukin 6 (IL-6) is known as a proinflammatory cytokine in chronic inflammatory diseases. In osteoarthritis, IL-6 released by joint tissue will bind to the soluble IL-6 receptor (IL-6R), leading to transsignaling [44]. As a consequence, the immune system is activated, whereby mononuclear cells like monocytes are recruited to the inflamed joint area [44]. IL-6 transsignaling will skew the differentiation of monocytes to macrophages through the upregulation of the macrophage colony-stimulating factor (M-CSF) receptor [45]. A significant increase of IL-6 and IL-8 has been observed in patients with osteoarthritis [46–51]. For instance, Favero et al. determined the inflammatory molecules produced from coculture of meniscus tissue and synovial membrane from patients with early-stage (*n* = 5) and end-stage (*n* = 5) osteoarthritis [47]. They demonstrated the presence of IL-6 and IL-8 in patients at both stages, but their levels were higher among the end-stage patients [47]. In osteoarthritis, IL-8 in the synovial fluid plays a role in recruiting neutrophils and activating them. The activated cells will secrete enzyme elastase to degrade type II collagen crosslinks and proteoglycan in the articular cartilage [52].

IL-37 is a member of the IL-1 family, and it is an anti-inflammatory cytokine [53]. Its level has been shown to elevate in osteoarthritis patients [53, 54]. IL-37 can reduce the synthesis of proinflammatory cytokines and catabolic enzymes by osteoarthritic chondrocytes and synoviocytes. Mabey et al. collected blood and synovial fluid samples from patients with osteoarthritis (*n* = 32) and healthy controls (*n* = 14) to determine inflammatory cytokine levels in patients with knee osteoarthritis. They demonstrated that IL-2, IL-4, IL-6, and IL-10 levels were higher in patients with osteoarthritis compared to the controls [55]. Besides, a study by Xia et al. also showed an increased IL-10 level in lymphocyte activation gene-3 negative (LAG-3^−^) regulatory T cells (Treg) from patients with knee osteoarthritis [16]. Conversely, He et al. demonstrated a decrease of IL-4 expression in articular cartilage from patients with osteoarthritis [56]. It may be due to the difference in the samples used between the signaling studies, whereby Mabey et al. used blood while He et al. used chondrocytes isolated from articular cartilage. The expression of IL-4 receptor had been shown to elevate in the serum of patients with osteoarthritis [57]. However, IL-4 was also shown to express at a decreased level in cartilage from patients with osteoarthritis compared to cartilage from healthy controls [58].

Other than the cytokines aforementioned, several other cytokines are involved in the pathogenesis of osteoarthritis. Some of the cytokines shown to increase in osteoarthritis are IL-18, TGF*β*1 [14], IL-1 receptor (IL-1R) [59], and IL-1 alpha (IL-1*α*) [23]. Shan et al. showed enhanced serum IL-21, IL-17A, and IFN-*γ* levels in patients with osteoarthritis compared to controls [15]. IL-21 also upregulates RANKL expression, thereby stimulating bone marrow stem cells to differentiate into mature osteoclasts [60]. The interactions between pro- and anti-inflammatory cytokines were explored using macrophage conditioned medium (CM). Upregulation of proinflammatory cytokines (IL-1b, IL-6, MMP13, and ADAMTS5) and downregulation of cartilage matrix components (aggrecan, type II collagen) were observed in human osteoarthritic cartilage explants cultured with CM of proinflammatory macrophages expressing IFN-*γ* and TNF-*α*. However, CM of anti-inflammatory macrophages expressing IL-4 or IL-10 did not suppress the stimulation effects of conditioned media of proinflammatory cytokine-stimulated explants [61].

## 5. The Role of Chemokines in Osteoarthritis

Chemokine is a subfamily of cytokine with low molecular weight. It is classified into four families, namely, CXC, CC, C, and CX3C families, depending on the position of cysteine (C) residues [62]. Chemokine functions as a chemoattractant, which directs the migration of immune cells to damaged or infected sites [62]. A study by Monasterio et al. revealed increased C-C motif ligand 5 (CCL5), CCL20, C-C motif receptor 5 (CCR5), and CCR7 in patients with temporomandibular joint osteoarthritis (TMJ-OA) [42]. These chemokines play a role in the recruitment of T helper cell type 1 (Th1), T helper cell type 17 (Th17), and T helper cell type 22 (Th22) to the affected joint in the study [42]. As a consequence, proinflammatory cytokines like IL-1*β*, IL-17, and IL-22 will be released in the joint and trigger the inflammation process.

In laboratory studies, chemokine receptor CXCR4 was shown by Sun et al. to reduce in chondrocyte culture induced with inflammation [29]. It is a specific receptor for stromal-derived-factor-1 (SDF-1), also called CXCL12. Raghu et al. revealed that CCL2 and its receptor, CCR2, were increased in a mouse model of osteoarthritis [63]. The study suggested that CCL2 is secreted by injured chondrocytes and synovial fibroblasts and it recruits CCR2-expressing monocytes to the damaged tissues.

CCL7 is a monocyte chemoattractant, which has been shown to increase in the synovial fluid of patients with osteoarthritis [41]. Production of CCL7 from synoviocytes is enhanced by IL-17 [41]. Patients with facet joint osteoarthritis (*n* = 48) also demonstrated elevated CCL4 and C-C Motif Chemokine Ligand 4 Like 2 (CCL4L2) compared to healthy controls (*n* = 10) [64]. The study showed that CCL4 and CCL4L2 expressions were involved in the canonical NF-*κ*B signaling pathway. Hou et al. discovered increased expression of CX3CL1 in synovial fibroblasts from patients with osteoarthritis [9]. CX3CL1 induced MMP-3 expression in a time-dependent and dose-dependent manner by reacting with its receptor, CX3CR1 [9]. Furthermore, Alaaeddine et al. showed enhanced expression of CCL20 and its receptor, CCR6, in cartilages compared to controls. This enhanced expression can further stimulate IL-6, MMP-1, and MMP-13 production [46].

Increased CCR3 and its ligand, CCL11, also known as eotaxin-1, have been detected in synovial cells from patients with osteoarthritis [21]. CCR3 is expressed by a few inflammatory cells such as T cells [65] and dendritic cells [66]. Besides, the study showed that CCL11 was able to stimulate the release of MMP-9 in synoviocytes from patients with osteoarthritis. Favero et al. observed the elevation of CCL21 and CCL5 in coculture of meniscus and synovial membrane from patients with osteoarthritis [47]. Conditioned media from osteoarthritis tissues like cartilage, IFP, meniscus, and synovium induced and enhanced production of CXCL8 and CCL21 in the synoviocyte cell line [67]. Several other studies showed an enhanced CCL2 level in patients with osteoarthritis [47, 63, 68]. CCL2 is also known as monocyte chemoattractant protein 1 (MCP-1). CCL2/CCR2 signaling will trigger monocyte trafficking into the inflamed joint area and, in turn, cause further inflammation. Arkestål et al. observed an increased CCR2 and a decreased CXCR3 expressions in the peripheral blood of patients with osteoarthritis compared to controls [69].

## 6. The Role of Matrix Metalloproteinases (MMPs) in Osteoarthritis

Matrix metalloproteinases (MMP) are a family of zinc-dependent enzymes well known for regulating the degradation of the extracellular matrix (ECM) through cleavage of peptide bond of the target proteins [70]. MMPs can be categorized into several groups, which are collagenases (MMP-1, MMP-13), gelatinases (MMP-2, MMP-9), stromelysins (MMP-3), metalloelastase (MMP-12), matrilysin (MMP-7), and membrane-type matrix metalloproteinases (MT-MMPs), according to its structure and substrates [71]. They can degrade ECM of the articular cartilage, which mainly consists of collagens and proteoglycans.

MMP-3, MMP-9, and MMP-13 levels increased in animals induced with osteoarthritis [18, 30, 33, 34, 63], but the TIMP-2 level was shown to decline [72]. Similarly, MMP-13, MMP-3, MMP-2, and MMP-9 increased in the cell line model of osteoarthritis [26, 28]. Furthermore, several studies have investigated the MMP-13 level in osteoarthritis patients, and consistent observation is obtained, whereby the MMP-13 level increased significantly in patients with osteoarthritis [30, 48, 59, 73–78]. MMP-13, as a collagenase, is responsible for the degradation of type II collagen [79], which is the main collagen type in articular cartilage [80]. The expression of MMP-13 increased through stimulation by CCL20 [46] and interferon regulatory factor-8 (IRF-8) in chondrocytes derived from patients with osteoarthritis [81]. Conversely, overexpression of IL-37 in chondrocytes decreased the MMP-13 level [53]. Two separate studies by Zhang et al. and Wu et al. showed that changes in microribonucleic acid (miRNA) expression in osteoarthritis could alter the MMP-13 level [35, 51]. They found that miR-502-5p and miR-454 enhanced the MMP-13 level. In addition, Ma et al. and Chen et al. demonstrated that advanced glycation end products (AGEs) remarkably induced MMP-13 expression in joint tissues from patients with osteoarthritis [23, 70].

MMP-1 is another known collagenase frequently found to be elevated in osteoarthritis [46, 53, 59, 82]. MMP-3, also known as stromelysin-1, cleaves type II collagen and aggrecan. Hou et al. showed that chemokine CX3CL1 induces MMP-3 production in a concentration-dependent and time-dependent manner using synovial fibroblasts from patients with osteoarthritis (OASFs) [9]. Other than that, several studies showed increased MMP-3 in osteoarthritis [47, 48, 53, 73, 78, 82, 83]. The induction of MMP-3 has been related to miR-149 and miR-454 expression in osteoarthritis [51, 84].

Expression of gelatinases such as MMP-2 (gelatinase A) and MMP-9 (gelatinase B) has been shown to associate with the pathogenesis of osteoarthritis. MMP-2 and MMP-9 are responsible to cleave ECM, cytokines, and chemokines, thus enhancing their activities [85]. MMP-2 level [14, 24, 86] and MMP-9 [21, 82] increase in inflamed synovial tissues compared to noninflamed tissues. MMP-10, named as stromelysin-2, also increases in early- and end-stage osteoarthritis. Favero et al. showed a significantly increased level of MMP-10 in the meniscus and synovial coculture compared to meniscus alone [47]. The synovium was collected from the suprapatellar pouch while meniscus was isolated from the inner superficial zone of osteoarthritis patients who underwent total knee replacement. The two tissues were cocultured using a transwell to separate it and allow interaction between the two tissues.

Tissue inhibitors of metalloproteinases (TIMPs) are MMP inhibitor. It is proposed that the imbalance between MMP and TIMP activities is linked to articular destruction in osteoarthritis [87]. TIMP-2, TIMP-3, and TIMP-4 are elevated in patients with osteoarthritis [47, 48], while TIMP-1 is decreased [59].

## 7. The Role of Signaling Pathways and Other Inflammatory Components in Osteoarthritis

The nuclear factor-kappa B (NF-*κ*B) transcription factor plays a central role in the pathogenesis of osteoarthritis [88]. It is triggered by proinflammatory cytokines and ECM degradation products [89]. The activated NF-*κ*B will modulate the expression of several cytokines, chemokines, and matrix-degrading enzymes, which explains its role in regulating catabolic events in osteoarthritis [26]. The NF-*κ*B signaling pathway begins with the activation of I*κ*B kinase (IKK), resulting in phosphorylation and degradation of I*κ*B*α* by the proteasome. Subsequently, p65 protein is released, phosphorylated, and translocated from the cytoplasm to the nucleus. These events activate the expression of several genes, such as MMP-13 and IL-6 [89]. Therefore, the phosphorylated p65 (p-p65) level was found to increase in osteoarthritis whereas the I*κ*B*α* level was decreased [22, 23, 31, 35, 84].

Enhanced p-p38, p-JNK, and p-ERK in osteoarthritis indicate the involvement of the mitogen-activated protein kinase (MAPK) signaling pathway [76]. MAPK is a mediator which regulates downstream expression of proinflammatory cytokines and MMPs [90]. It also acts as a pain mediator [90]. This pathway could be a potential avenue for new drug discovery to halt the progression of osteoarthritis. It begins when proinflammatory cytokines and growth factors bind to their respective receptors on the cell membrane. These act as upstream activators and cause intracellular MAP kinases (MKKs) to phosphorylate specific MAP kinases. MKK1 and 2 will activate ERK1 and 2 and MKK3 and 6 responsible for p38 phosphorylation while MKK4 and 7 phosphorylate JNK1 and 2. Activated MAP kinases in turn activate other protein kinases and transcriptional regulatory proteins which lead to the upregulation of certain inflammatory genes such as MMPs, IL-1, and TNF-*α*. These cytokines can then maintain JNK activation and cause more cytokine and MMP production [90].

PI3K/AKT signaling is known to be activated by cytokines like IL-1*β* when it binds to its cell surface receptor. Upon stimulation, membrane protein PI3K induces phosphorylation of AKT which has shown to have a synergistic effect on NF-*κ*B signaling. Activation of the PI3K/AKT pathway will enhance production of MMPs by cells, for example, chondrocytes [91].

The nitric oxide (inducible nitric oxide synthase (iNOS), nitric oxide (NO)) and prostacyclin pathway (cyclooxygenase-2 (COX-2), prostaglandin E2 (PGE2)) are also integral to the pathogenesis of osteoarthritis. IL-1*β* upregulates both iNOS and COX-2 in osteoarthritis, leading to increased production of NO [92] and PGE2 [93], respectively. Elevated NO will inhibit the synthesis of collagen type II (Col2) and proteoglycan [92]. Besides, enhanced PGE2 inhibits chondrocyte proliferation and reduces ECM synthesis [93]. In addition, IL-1*β* also stimulates A Disintegrin and Metalloproteinase with Thrombospondin motif (ADAMTS-5) production, an aggrecanase which causes aggrecan degradation [94]. iNOS, NO, COX-2, PGE2, ADAMTS-5, ADAMTS-4, and VEGF were found to increase in animals with osteoarthritis, leading to enhanced inflammatory factor production and ECM degradation [30, 34].

There are other factors influencing the progression of osteoarthritis. The expression of runt-related transcription factor 2 (Runx2), an osteogenic transcriptional activator, is enhanced in osteoarthritis [95]. Terauchi et al. found elevated Runx2 in STR/OrtCrlj mouse osteoarthritis model [95]. Runx2 was shown to promote MMP-13 expression [95]. The expression of karyopherin alpha 2 (KPNA2), which regulates delivery of p65 to the nucleus, also increased in osteoarthritis [96]. Bromodomain-containing protein 4 (BRD4) played a role in the NF-*κ*B signaling pathway [28]. Inhibition of BRD4 will suppress IL-1*β*-induced expression of proinflammatory cytokines and phosphorylation of p65. The BRD4 level was increased in a C57BL/6 mouse model of osteoarthritis [28]. Nerve growth factor (NGF) had been shown by Blaney Davidson et al. to increase in bovine chondrocytes treated with TGF-*β*1 and IL-1*β* [97]. TGF-*β*1-induced NGF expression was found to be dependent on an activin receptor-like kinase 5-Smad2/3 (ALK5-Smad2/3) signaling pathway as blockage of this pathway can prevent the expression. ALK5 is a type of transmembrane receptor of TGF-*β*. Once it activates, it will trigger downstream signaling cascades via the Smad-dependent pathway [98]. In a recent study using cartilage explants cultured with osteoarthritic synovium-CM or IL-1*β*, the TGF-*β*/Smad2/3P pathway that is protective against mechanical loading was diminished [99], which could lead to further destruction of the cartilage. Moreover, the complement system components [100] and soluble macrophage biomarkers (CD163 and CD14) [101] are also shown to be upregulated in osteoarthritis.

**Figure 1:**
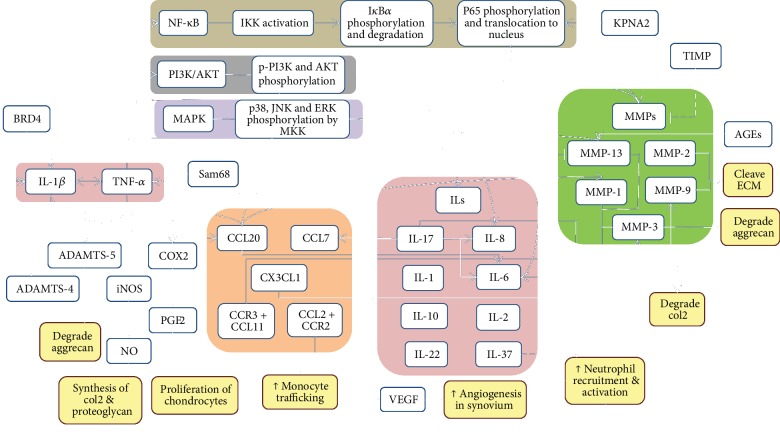
The signaling pathways (brown, grey, and purple box) involved and interactions between cytokines (pink box), chemokines (beige box), matrix metalloproteinases (green box), and other proteins in osteoarthritis. IL-1*β* and TNF-*α* (pink box) produced by cells; for example, activated chondrocytes, synoviocytes, and mononuclear cells are among the early mediators in the inflammatory cascades. Continuous lines indicate stimulation while dotted lines indicate inhibition of the downstream molecule or activity. The outcomes of the molecule interactions are shown in yellow boxes.

Overall, the integrated regulation of cytokines, chemokines, MMPs, and the signaling pathway is summarized in Figure 1.

## 8. The Role of miRNA in Osteoarthritis

**Figure 2:**
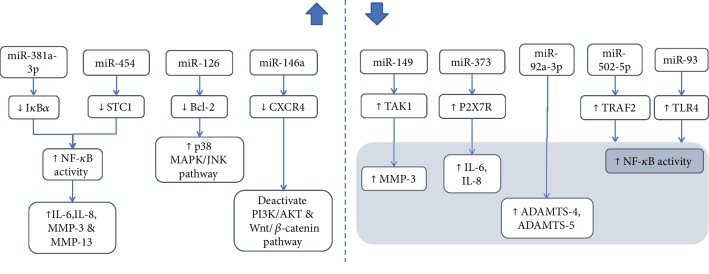
Alteration of miRNA levels in osteoarthritis and its downstream influences.

MicroRNAs (miRNAs) are short, endogenous noncoding RNAs functioning to regulate posttranscriptional gene expression by binding to the 3′untranslated region (3′UTR) of target genes [31, 35]. Once bound to target genes, they can block the translation process or decrease the stability of the messenger RNAs (mRNAs) [102]. Several microarray and database analyses have shown the involvement of miRNAs in osteoarthritis (Figure 2) [103].

Laboratory studies showed that miR-146a [29] and miR-126 [27] were upregulated in chondrocytes induced with inflammation with lipopolysaccharide (LPS) and IL-1*β*, respectively. miR-146a targets CXCR4 and downregulated its expression. Besides, upregulation of miR-126 in IL-1*β*-treated chondrocytes downregulated B-cell lymphoma 2 (Bcl-2) expression. miR-381a-3p was found to be elevated, and miR-93 was decreased in a rodent model of osteoarthritis [31, 32].

Recent human studies highlighted that miR-381a-3p [31] and miR-454 [51] were upregulated in osteoarthritis. Xia et al. discovered that nuclear factor of kappa light polypeptide gene enhancer in B-cell inhibitor alpha (I*κ*B*α*) is a target gene of miR-381a-3p [31]. Hence, upregulation of miR-381a-3p will decrease I*κ*B*α* expression and further enhance the activation of NF-*κ*B. Wu et al. showed that miR-454 overexpression in osteoarthritis promoted the proliferation of synovial fibroblasts and increased inflammatory factors [51].

On the other hand, Chen et al. showed that miR-149 bound to transforming growth factor- (TGF-) 1-activating kinase 1 (TAK1) 3′UTR to regulate its expression [84]. TAK1 expression increased when miR-149 decreased in osteoarthritis. NF-*κ*B is the downstream signaling pathway of TAK1; thus, upregulated TAK1 causes increased activation of NF-*κ*B in osteoarthritis [104]. Zhang et al. demonstrated that miR-373 decreased in osteoarthritis and it targeted P2X7 receptor (P2X7R) [49]. P2X7R expression was enhanced in osteoarthritis; this led to increased chondrocyte proliferation and release of inflammatory factors such as IL-6 and IL-8. In addition, P2X7R is adenosine triphosphate- (ATP-) gated plasma membrane ion channel which is involved in IL-1*β* maturation and its release from activated immune cells [105]. Damaged cells released ATP which led to activation of P2X7R and further release of inflammatory cytokines [106]. Mao et al. revealed that miR92a-3p directly targeted 3′UTR of ADAMTS-4 and ADAMTS-5 mRNAs [107]. Zhang et al. showed that miR-502-5p targeted 3′UTR of TNF receptor-associated factor 2 (TRAF2) to inhibit its expression [35]. Since these miRNAs target mRNAs which contribute to the development of osteoarthritis, they could be potential drug targets to treat osteoarthritis.

### 8.1. Future Research Area

The understanding of mechanisms involved in the pathogenesis of osteoarthritis is very important. It is the basis of rational drug development as currently, most pharmacotherapies for osteoarthritis are symptomatic. For example, the integral role of IL-1*β* in the pathogenesis of osteoarthritis can be targeted to prevent further degradation of the joint tissue. IL-1 receptor antagonist has been developed, and it showed promising results in animal studies. However, its effects on humans require further studies [108]. Besides, IL-6 is well known for its contribution to progression of osteoarthritis. IL-6 or its receptor such as soluble IL-6 receptor can be a target for drug development. The recently discovered functional role of miRNAs in regulating joint homeostasis should also be explored, and they are another avenue for intervention. Circular RNAs (circRNAs), a single-stranded, noncoding regulatory RNA, can function as miRNA sponges and inhibit miRNA activity. It provides a possibility to be targeted as a therapeutic strategy.

The use of inflammatory markers to predict disease progression and treatment efficacy should also be explored. Currently, the evaluation of patients' conditions and improvements is based on subjective instruments like Western Ontario and McMaster Universities Osteoarthritis Index (WOMAC) and radiographic findings. Future studies should attempt to validate the correlation of these instruments with inflammatory markers to provide a more objective measurement of disease progression. Established biomarkers of osteoarthritis progression such as urinary type II collagen degradation (uCTX-II) are shown to associate with incidence and progression of radiographic osteoarthritis [109].

## 9. Conclusion

Inflammation plays an integral role in the pathogenesis of osteoarthritis. Various molecules released by the chondrocytes and synoviocytes and infiltrating immune cells, such as cytokines, chemokines, and MMPs, are involved in regulating the joint anabolism and catabolism process. Their expressions are in turn governed by NF*κ*B, MAPK, PI3K/AKT, prostacyclin, and nitric oxide pathways. The involvement of these molecules and signaling pathways is well-established in cellular, animal, and human studies. However, more studies are required to link and explore the connection between all these molecules involved. This review article collates the latest evidence on the relationship between inflammation and osteoarthritis to provide a better understanding on the pathogenesis of osteoarthritis. These pathways should be exploited as potential targets for drug intervention as currently pharmacotherapies targeting the underlying mechanism of osteoarthritis are still lacking in the market.

## Figures and Tables

**Table 1:** Molecular changes in humans with osteoarthritis.

Authors (year)	Subjects' characteristics	Sample	Molecules and cells involved					
			Cytokines	Chemokines	Matrix metalloproteinases (MMPs)	Immune cells	Other proteins	miRNA
Hou et al. (2017) [9]	OA patients, *n* = 10 Non-OA patients, *n* = 8	Synovial tissue		↑ CX3CL1	↑ MMP-3			
Deligne et al. (2015) [14]	OA patients, *n* = 32, mean age 70.3 ± 9.8 years	Synovial tissues	↑ IL-1*β*, IL-6, IL-8, IL-18, IL17, IL-22, and TGF*β*1		↑ MMP-2	↑ Leukocyte (macrophages, T-lymphocytes, B-lymphocytes, neutrophils) infiltrates	↑ MPO	
Shan et al. (2017) [15]	OA patients, *n* = 40, median age 65 years Healthy control, *n* = 13, median age 61 years	Fasting venous blood	↑ IL-21, IFN-*γ*, and IL-17A			↑ PD-1+CXCR5+CD4+ T cells, ICOS+CXCR5+CD4+ T cells, and IL-21+CXCR5+CD4+ T cells	↑ CRP	
Xia et al. (2017) [16]	OA patients, *n* = 16 Healthy control, *n* = 16	Blood Synovial fluid	↑ IL-10 and TGF-*β*			↑ LAG-3+ Treg cells		
Min et al. (2017) [20]	Knee OA patients, *n* = 148, mean age 68.0 years Healthy control, *n* = 101, mean age 57.7 years	Fasting blood	↑ TNF-*α*				↑ Serum OPG ↓ DKK1	
Chang et al. (2015) [21]	OA patients, *n* = 15, mean age 60.7 ± 4.4 years	Peripheral blood Articular synovial membrane	↑ TNF-*α* and IL-1*β*	↑ CCR3 and eotaxin-1(CCL11)	↑ MMP-9			
Ni et al. (2015) [22]	OA patients, *n* = 58, median age 66 years Healthy control, *n* = 30, median age 60 years	Blood Synovial fluid Synovial tissues	↑ TNF-*α*, IL-1*β*, and IL-6				↑ FSTL1, p-p65, and p-I*κ*B*α* ↓ p53 and p21	
Ma et al. (2015) [23]	OA patients, *n* = 6, mean age 31.2 ± 2.91 years	Articular cartilages	↑ IL-1*α*, IL-1*β*, and TNF-*α*		↑ MMP-13		↑ p65 nuclear level ↓ I*κ*B*α* degradation	
Ding et al. (2015) [24]	OA patients, *n* = 25, mean age 66.6 years	Synovial tissues			↑ MMP-2		↑ Cadherin-11	
Xu et al. (2015) [26]	OA patients, *n* = 5 Normal control, *n* = 3	Articular cartilage					↑ Sam68 expression	
Jiang et al. (2017) [28]	OA patients, *n* = 20	Articular cartilages					↑ BRD4	
Qu et al. (2018) [30]		Articular cartilage	↑ IL-6 and TNF-*α*		↑ MMP-13		↓ Ghrelin, ACAN, col2, sox-9, and GAG ↑ ADAMTS-5 and iNOS	
Xia et al. (2016) [31]	OA patients, *n* = 10 Normal control, *n* = 10	Blood Articular cartilage Synovial tissues	↑ TNF*α*, IL-6, and IL8				↑ COX-2, iNOS, and p-p65 ↓ IkB*α*	↑ miR-381a-3p
Zhang et al. (2016) [35]	OA patients, *n* = 20 Normal control, *n* = 20	Articular cartilages	↑ IL-6, TNF-*α*		↑ MMP-13		↓ ACAN, col2, and Ik-B*α* expression ↑ TRAF2 and p-p65 expression	↓ miR-502-5p
Snelling et al. (2017) [41]	OA patients, *n* = 152, mean age 73 ± 9 years	Blood Synovial fluid	14 patients had detectable IL-17 in SF with ↑ IL-6	↑ CCL7 in the 14 patients			↑ Leptin, resistin, and NGF	
Monasterio et al. (2018) [42]	TMJ-OA patients, *n* = 4, mean age 53.6 ± 26.6 years Control: DDWR patients, *n* = 2, mean age 24.5 ± 2.1 years	Synovial fluid	↑ IL-17, IL-1*β*, IL-22, and RANKL	↑ CCL5, CCL20, CCR5, and CCR7				
Alaaeddine et al. (2015) [46]	OA patients, *n* = 21, mean age 67 ± 19 years Non-OA donor, *n* = 8, mean age 30 ± 27 years	Knee cartilage	↑ IL-6 by CCL20	↑ CCL20 and CCR6	↑ MMP-1 and MMP-13 by CCL20		↑ PGE2 and proteoglycan by CCL20 ↑ ADAMTS-5 and col10 mRNA expression	
Favero et al. (2018) [47]	Early-stage OA patients, *n* = 5, median age 34 years End-stage OA patients, *n* = 5, median age 62 years	Synovial tissues	↑ IL-6 and IL-8	↑ CCL2, CCL21, and CCL5 (RANTES)	↑ MMP-3 and MMP-10		↑ TIMP-2, TIMP-4 ↑ GAG and ACAN CS846 epitope	
Capsoni et al. (2015) [48]		Articular cartilage	↑ IL-6 and IL-8		↑ MMP-3 and MMP-13		↑ NO, iNOS, TIMP-3, and TIMP-4	
Zhang et al. (2017) [49]	OA patients, *n* = 11, average age 49 years Healthy control, *n* = 12, average age 42 years	Knee articular cartilage Blood	↑ IL-6, IL-8				↑ P2X7R	↓ miR-373
Böhm et al. (2016) [50]	OA patients, *n* = 22, mean age 66.7 years	Synovial tissue	↑ IL-6 and IL-8					
Wu et al. (2018) [51]	OA patients, *n* = 40, mean age 43 years Normal control, *n* = 10, mean age 39 years	Synovial tissue	↑ IL-6, IL-8		↑ MMP-3, MMP-13		↓ STC1	↑ miR-454
van Geffen et al. (2016) [53]	OA patients, *n* = 8	Articular cartilage	↑ IL37, IL-6, and IL-8		↑ MMP-1, MMP-3, and MMP-13		↑ ADAMTS5	
Ding et al. (2017) [54]	OA patients, *n* = 72, mean age 63.89 ± 14.34 years, and disease duration 4.56 ± 3.9 years Healthy control, *n* = 40, mean age 62.32 ± 14.15 years	Blood Synovial fluid Synovial tissues	↑ IL-37, IL-1*β*, TNF-*α*, and IL-6					
Mabey et al. (2016) [55]	OA patients, *n* = 32, mean age 64.6 ± 6.2 years Healthy control, *n* = 14, mean age 63.9 ± 6.4 years	Blood samples Synovial fluid	↑ IL-2, IL-4, IL-6, and IL-10					
He et al. (2017) [56]	OA patients, *n* = 12, mean age 65.8 ± 3.5 years	Articular cartilage	↑ IL-6, TNF-*α*, and IFN-*γ* ↓ IL-4				↑ SOCS1, caspase 9, Bax, Bcl-2, and iNOS	
Sun et al. (2015) [59]		Articular cartilage	↑ IL-1R and TNF-*α*		↑ MMP-1 and MMP-13		↑ PARP-1, iNOS, and p-p65 ↓ TIMP-1	
Raghu et al. (2017) [63]	OA patients, *n* = 35 Normal control, *n* = 37	Synovial tissue Synovial fluid		↑ CCL2				
Chen et al. (2018) [64]	Facet joint OA patients, *n* = 48, mean age 64 ± 1.7 years Healthy control, *n* = 10, mean age 25 ± 1.2 years	Facet joint tissues		↑ CCL4 and CCL4L2			↑ DKK2	
Belluzzi et al. (2018) [67]	OA patients, *n* = 5, median age 68 years	Cartilage Synovial membrane tissues Meniscus IFP	↑ IL-6, IL-1*β*	↑ CXCL8, CCL21	↑ MMP-10			
Huang et al. (2016) [68]		Synovial tissues	↑ IL-6, IL-1*β*, and TNF-*α*	↑ MCP-1 (CCL2)			↑ NO, PGE2, iNOS, COX-2, VCAM-1, ICAM-1, ET-1, TF, and p-IkB	
Arkestål et al. (2018) [69]	OA patients, *n* = 7 Healthy control, *n* = 9	Peripheral blood Bone marrow		↑ CCR2 ↓ CXCR3				
Chen et al. (2015) [70]		Synovial tissues	↑ IL-6		↑ MMP-13		↑ COX-2, PGE2, VEGF, p-IKK *α*/*β*, p-IkB*α*, and p-p65 expression	
Zeng et al. (2019) [73]	OA patients, *n* = 12 Patients with other joint diseases, *n* = 12	Articular cartilage	↑ TNF-*α* and IL-6		↑ MMP-3 and MMP-13		↑ FOXM1, iNOS, COX-2, NO, PGE2, and p-p65	
Xia et al. (2017) [74]		Cartilage tissues	↑ IL-6 and TNF-*α*		↑ MMP-13		↑ PRMT1, ADAMTS-5, NO, PGE2, iNOS, COX-2, SHH, Gli-1, and Patch 1 ↓ ACAN and COL2A1	
Burguera et al. (2014) [75]	OA patients, *n* = 13, mean age 77.5 ± 10 years	Articular cartilages	↑ IL-6		↑ MMP-13		↑ COX2, PGE-2, and nitrite	
Peng et al. (2017) [76]	OA patients, *n* = 5, mean age 65.2 ± 3.2 years Normal control, *n* = 3, mean age 31.0 ± 5.9 years	Synovial tissues			↑ MMP-13		↓ DUSP1 ↑ p-p38, p-JNK, and COX-2	
Ma et al. (2018) [77]	OA patients, *n* = 30	Knee cartilage	↑ IL-8		↑ MMP-13		↑ p-PKR, p-PKC, COX-2, ROS, p-ERK, and p65 activity ↓ SOD, catalase, and PPAR-*γ*	
Haneda et al. (2018) [78]	OA patients, *n* = 31, average age 76.4 years Normal control, *n* = 12, average age 85.1 years	Cartilage tissues			↑ MMP-3, MMP-13		↑ AQP1, ADAMTS-4, and ADAMTS-5 ↓ COL2A1 and ACAN expression	
Yang et al. (2017) [81]	OA patients, *n* = 8 Normal control, *n* = 12	Articular cartilage			↑ MMP-13		↑ IRF-8	
Fu et al. (2016) [82]	Normal donors	Articular cartilage			↑ MMP-1, MMP-3, and MMP-9		↑ NO, PGE2, iNOS, COX-2, ADAMTS-4, ADAMTS-5, HMGB1, and TLR4 expression	
Chou et al. (2018) [83]	OA patients, *n* = 20 for cartilage samples, mean age 66.6 ± 9.9 years; *n* = 25 for synovial fluid samples, mean age 63.6 ± 15.7 years	Knee articular cartilage Synovial fluid	↑ IL-6		↑ MMP-3		↑ TSG-6, TIMP1, and VEGF	
Chen et al. (2018) [84]	OA patients, *n* = 20, mean age 62 ± 9.2 years Healthy donor, *n* = 20, mean age 55.2 ± 8.64 years	Articular cartilage	↑ IL-6		↑ MMP-3		↑ TAK1, nitrite, PGE2, and p-p65 ↓ IkB*α*	↓ miR-149
Alunno et al. (2017) [86]	OA patients, *n* = 24	Synovial fluid FLS			↑ MMP-2			
Gui et al. (2017) [88]	OA patients, *n* = 10, mean age 63.4 years	Articular cartilages					↑ SOCS3, NF-*κ*B, and COX2	
Terauchi et al. (2016) [95]	OA patients, *n* = 5	Articular cartilage					↑ Runx2	
Tao et al. (2015) [96]	OA patients, *n* = 10, mean age 50 ± 10 years Healthy control, *n* = 10, mean age 55 ± 10 years	Articular cartilage					↑ KPNA2	
Struglics et al. (2016) [100]	OA patients, *n* = 24, median age 64 years	Synovial fluid	↑ IL-1*β*, IL-6, IL-8, and TNF-*α*				↑ C4d, C3bBbP, and sTCC	
Daghestani et al. (2015) [101]	OA patients, *n* = 159, mean age 63.7 ± 11.8 years	Synovial fluid Blood					↑ CD163 and CD14	
Mao et al. (2017) [107]	OA patients, *n* = 8, mean age 65.8 ± 2.24 years Healthy donor, *n* = 8, mean age 64.4 ± 2.86 years Normal donors for bone marrow, *n* = 6, mean age 37 years	Bone marrow Cartilage tissues					↑ ADAMTS-4 and ADAMTS-5 ↓ ACAN	↓ miR-92a-3p

**Table 2:** Molecular changes in animal model of osteoarthritis.

Authors (year)	Animal model	Osteoarthritis intervention	Samples	Findings
Schmidli et al. (2018) [18]	Dogs (*n* = 59): diseased group (*n* = 36) and control group (*n* = 23)	Diseased dogs with canine cruciate ligament disease used	Infrapatellar fat pad Subcutaneous adipose tissue Synovial fluid	↑ IL-1*β*, IL-6, IL-10, MMP-1, MMP-3, MMP-13, and TNF-*α* ↑ T cells, macrophages
Xu et al. (2015) [26]	Male Sprague-Dawley (SD) rats (*n* = 20), aged 40–50 days: normal group (*n* = 10) and 8-week group (*n* = 10)	Meniscal/ligamentous injury (MLI) modeling in knee joint	Articular cartilage	↑ Sam68 expression
Jiang et al. (2017) [28]	C57BL/6 mice (*n* = 20)	ACLT	Knee joints	↑ BRD4, HMGB1, and p-p65
Qu et al. (2018) [30]	Male C57BL/6 mice	Surgically induced destabilization of medial meniscus (DMM)	Knee joints	↑ MMP-13, TNF-*α*, iNOS, ADAMTS-5, IL-6, COX-2, NF-*κ*B2, and p-I*κ*B*α*
Xia et al. (2016) [31]	SD rats	MIA injection (0.5 mg) for 4 weeks	Blood	↑ miR-381a-3p, TNF*α*, COX-2, iNOS,IL-6, IL-8, and p-p65 ↓ I*κ*B*α*
Ding et al. (2019) [32]	Male C57BL/6 mice (*n* = 36)	Medial meniscal tear surgery	Articular cartilages of medial tibial plateau Synovial fluid	↓ miR-93 ↑ TNF-*α*, IL-1*β*, IL-6, TLR4, p-p65, and p-I*κ*B*α*
Hu et al. (2016) [33]	Male Wistar rats (MIA group, *n* = 30; control, *n* = 15)	Intra-articular MIA injection (5 mg/kg)	Blood	↑ P2X7R, TNF-*α*, IL-6, IL-1*β*, MMP-13, SP (substance P), and PGE2 ↑ IKK*α*, IKK*β*, I*κ*B*α*, p65, and all of their phosphorylated forms
Li et al. (2018) [34]	Female SD rats (*n* = 36)	Intra-articular MIA injection (0.2 mg/rat) for 10 days	Synovial fibroblasts Blood	↑ TNF-*α*, IL-1*β*, IL-17a, IL-8, MMP-3, MMP-9, VEGF, ADAMTS-4, p-PI3K, and p-AKT
Raghu et al. (2017) [63]	C57BL/6J mice	Destabilization of medial meniscus (DMM)	Knee joints	↑ CCL2, CCR2, MMP-13, MMP-6, and ADAMTS-4
Adler et al. (2017) [72]	Mixed-breed dogs (*n* = 4)	TGF-1*β* (1 or 10 ng/ml) with or without IL-1*β* (10 ng/ml)	Stifle joints' cartilage	↑ MMP-3, iNOS, NO, COX-2, and PGE ↓ TIMP-2
Terauchi et al. (2016) [95]	Male STR/OrtCrlj mice (*n* = 24)		Articular cartilage with subchondral bone	↑ Runx2 expression
Blaney Davidson et al. (2015) [97]	Cow	TGF-*β*1 (1 and 10 ng/ml), IL-1*β* (1 or 10 ng/ml)	Metacarpal joint's cartilage	↑ NGF
Alquraini et al. (2017) [110]	Male Prg4+/+ and Prg4-/- mice	rhPRG4 (100 *μ*g/ml), CD44 Ab (1.25 *μ*g/ml), or combination of rhPRG4 and CD44 Ab for 48 hours	Synovial tissues	↑ NF-*κ*B p50 and p65 in Prg4-/- compared to Prg4+/+

**Table 3:** Molecular changes in cellular model of osteoarthritis.

Authors (year)	Cell line	Treatment	Findings
Xu et al. (2015) [26]	SW1353	100 ng/ml human TNF-*α* for 0, 6, 12, 24, 36, and 48 h	↑ Cleaved caspase-3, cleaved PARP, Sam68, MMP-13, ADAMTS-5, iNOS, and IL-6 ↓ I*κ*B*α*, ↑ p-p65
Yu et al. (2018) [27]	CHON-001 (human chondrocyte cell)	IL-1*β* (0.1 ng/ml, 2 ng/ml, 5 ng/ml, and 10 ng/ml)	↑ miR-126, IL-6, IL-8, and TNF-*α*
Jiang et al. (2017) [28]	SW1353	10 ng/ml IL-1*β*	↑ IL-6, IL-8, IL-10, TNF-*α*, MMP-2, MMP-3, MMP-9, MMP-13, BRD4, HMGB1, and nuclear p65
Sun et al. (2017) [29]	ATDC5 (murine articular chondrocyte)	LPS (0, 1, 5, and 10 *μ*g/ml) for 6 hrs	↑ miR-146a, IL-1*β*, IL-6, IL-8, and TNF-*α* ↓ CXCR4
Tao et al. (2015) [96]	SW1353 (human chondrosarcoma cells)	10 ng/ml IL-1*β* for 0, 12, 24, 36, and 48 hrs respectively	↑ KPNA2, MMP-13, and ADAMTS-5 ↑ Nuclear p65, p-p65
Blaney Davidson et al. (2015) [97]	H4 (murine chondrocyte), G6 (human chondrocyte)	IL-1*β* (1 or 10 ng/ml) or TGF-*β*1 (0.1, 1, or 10 ng/ml)	↑ NGF

## Abbreviations

ACAN: Aggrecan
ACLT: Anterior cruciate ligament transection
ADAMTS: A Disintegrin and Metalloproteinase with Thrombospondin motifs
AGEs: Advanced glycation end products
AKT: Protein kinase B
ALK5: Activin receptor-like kinase 5
AQP1: Aquaporin 1
ATP: Adenosine triphosphate
BCL-2: B-cell lymphoma 2
BRD4: Bromodomain-containing protein 4
CCL: C-C motif ligand
CCR: C-C motif receptor
CRP: C reactive protein
CXCR: C-X-C chemokine receptor
col10: Collagen type X
col2: Collagen type II
COX-2: Cyclooxygenase 2
DDWR: Disk displacement with reduction
DKK: Dickkopf WNT signaling pathway inhibitor
DMM: Destabilization of medial meniscus
DUSP1: Dual specificity protein phosphatase 1
ECM: Extracellular matrix
ERK: Extracellular signal-regulated kinase
ET-1: Endothelin-1
FLS: Fibroblast-like synoviocytes
FOXM1: Forkhead box M1
FSTL1: Follistatin-like protein 1
GAG: Glycosaminoglycan
HMGB1: High mobility group box 1
ICAM-1: Intracellular adhesion molecule-1
ICOS: Inducible costimulator
IFP: Infrapatellar fat pad
IFN: Interferon
IKK: I*κ*B kinase
IL: Interleukin
iNOS: Inducible nitric oxide synthase
IRF-8: Interferon regulatory factor-8
JNK: c-Jun N-terminal kinase
KPNA2: Karyopherin alpha 2
LAG: Lymphocyte activation gene
MAPK: Mitogen-activated protein kinase
MCP-1: Monocyte chemoattractant protein 1
M-CSF: Macrophage colony-stimulating factor
MIA: Monosodium iodoacetate
MKKs: MAP kinase kinases
MMPs: Matrix metalloproteinases
MPO: Myeloperoxidase
mRNA: Messenger ribonucleic acid
miRNA: Microribonucleic acid
NF-*κ*B: Nuclear factor kappa-light-chain-enhancer of activated B cells
NGF: Nerve growth factor
NO: Nitric oxide
OA: Osteoarthritis
OPG: Osteoprotegerin
PARP-1: Poly [ADP-ribose] polymerase 1
PD-1: Programmed cell death 1
PGE2: Prostaglandin E2
PI3K: Phosphoinositide 3-kinase
PKC: Protein kinase C
PKR: Protein kinase R
PPAR-*γ*: Peroxisome proliferator-activated receptor gamma
PRMT1: Protein arginine N-methyltransferase 1
RANKL: Receptor activator of nuclear factor kappa-*Β* ligand
ROS: Reactive oxygen species
Runx2: Runt-related transcription factor 2
SD: Sprague-Dawley
SOCS1: Suppressor of cytokine signaling 1
SOD: Superoxide dismutase
STC1: Stanniocalcin-1
sTCC: Soluble terminal complement complex
TAK1: Transforming growth factor-1-activating kinase 1
TF: Tissue factor
TGF: Transforming growth factor
Th: T helper cell
TIMP: Tissue inhibitor of metalloproteinase
TLR4: Toll-like receptor 4
TMJ: Temporomandibular joint
TNF: Tumor necrosis factor
TRAF2: TNF receptor-associated factor 2
Treg: Regulatory T cells
TSG-6: TNF-stimulated gene-6
uCTX-II: Urinary type II collagen degradation
UTR: Untranslated region
VCAM-1: Vascular cell adhesion molecule-1
VEGF: Vascular endothelial growth factor
WOMAC: Western Ontario and McMaster Universities Osteoarthritis Index.
